# Coverage and Awareness of and Compliance with Mass Drug Administration for Elimination of Lymphatic Filariasis in Burdwan District, West Bengal, India

**DOI:** 10.3329/jhpn.v31i2.16380

**Published:** 2013-06

**Authors:** Rabindra Nath Roy, Aditya Prasad Sarkar, Raghunath Misra, Amitava Chakroborty, Tusar Kanti Mondal, Kanad Bag

**Affiliations:** Community Medicine, Burdwan Medical College, India

**Keywords:** Compliance, Coverage, Lymphatic filariasis, Mass drug administration, India

## Abstract

India adopted WHO's strategy of repeated rounds of mass drug administration (MDA) with diethylcarbamazine to eliminate lymphatic filariasis. The present study attempted to assess the coverage and awareness of and compliance with MDA for elimination of lymphatic filariasis in Burdwan district of India, following MDA round in July 2010. A cross-sectional study was conducted among the four randomly-selected clusters in the district of Burdwan, West Bengal, India, covering 603 individuals from 154 households, using a predesigned pretested schedule. The drug distribution coverage, compliance, and effective coverage were 48.76 %, 70.07%, and 34.16% respectively. Only 41.4% of the study population was aware of the MDA activity. This evaluation study noted that MDA is restricted to tablet distribution only. There is an urgent need to improve compliance with drug intake through strengthening of the awareness programme involving both government health workers and community volunteers.

## INTRODUCTION

Lymphatic filariasis (LF) is one of the most debilitating and disfiguring scourges among all diseases. Globally, 1.3 billion people are estimated to be at risk of infection, and some 120 million people are infected in 83 countries. The South-East Asia Region (SEAR) accounts for about 65% of the global population at risk and 50% of the infected people. Nine of the 11 countries in the region are known to be endemic for filariasis (1).

Filariasis has been a major public-health problem in India. The disease is reported to be endemic in 250 districts in 20 states and union territories (UTs) of India. About 600 million people are at risk of lymphatic filariasis in these districts. Indigenous cases of lymphatic filariasis have been reported from most of the states, including West Bengal. However, some north-western states/UTs, namely Jammu and Kashmir, Himachal Pradesh, Punjab, Haryana, Chandigarh, Rajasthan, Delhi, Uttaranchal and north-eastern states, namely Sikkim, Arunachal Pradesh, Nagaland, Meghalaya, Mizoram, Manipur, and Tripura, are stated to be free from indigenously-acquired filarial infection (2).

The National Health Policy (2002) has envisaged elimination of lymphatic filariasis in India by 2015 (3). Elimination of LF means cessation of LF as a public-health problem, when the microfilaria (Mf) rate is less than one percent among the population in all areas of an endemic country and the children born after elimination of LF are free from circulating antigenaemia. Absence of antigenaemia among children is considered an evidence of the absence of transmission and new infection (4,5). The strategy for achieving the goal of LF elimination is by annual mass drug administration (MDA) with diethylcarbamazine (DEC) to the entire population at risk and morbidity management of lymphedema, along with the other vector management strategies (2). MDA, in combination with other techniques, has already eliminated filariasis from Japan and South Korea and markedly reduced the transmission in China (3). The Government of India launched administration of annual single-dose DEC in 2004 and proposed the day of administration of MDA to be observed as National Filaria Day (NFD) every year in the endemic districts. Under this programme, a single-dose antifilarial drug DEC is administered in the dose of 6 mg/kg of body-weight to inhabitants in filaria-endemic areas, excluding children below 2 years of age, pregnant women, and seriously-ill persons. A sustainable high coverage (>85%) in endemic areas for 5 years or more is required to achieve the interruption of transmission and elimination of the disease (2). Rate of coverage and consumption compliance are the most crucial factors in the success of MDA strategy (4).

Adverse drug reactions may decrease the compliance with drug consumption at the community level. It is of great advantage that the side-effects following DEC administration are mild or absent when the drug is given in daily doses of 6 mg/kg or less. The DEC consumption may be associated with minor side-effects in 1-10% of the treated persons, particularly among the carriers of microfilariae. The community is made aware of the temporary side-effects that may occur in the population who may be carriers of microfilariae. The non-specific drug-related reactions include: headache, anorexia, nausea, abdominal pain, vomiting, dizziness, weakness, or lethargy. These symptoms begin within 1-2 hour(s) of taking the drug and persist for a few hours. Specific parasite-related allergic reactions due to destruction of microfilariae and adult worms include: fever, local inflammations around dead worms, and pruritus. Most of these side-effects are self-limiting (4).

Different field studies indicated suboptimal performance in the coverage of annual DEC consumption by eligible population (6). Health education is instrumental for the awareness-generation and active participation of the community and forms an integral part of the elimination strategy. The knowledge gap with regard to the disease and prevailing attitudes toward the programme may be the causes of poor compliance with the consumption of drugs. WHO has recommended periodic evaluation of the status of implementation of MDA activities by independent experts.

With this background, the present study attempted to assess the coverage and community awareness of and compliance with MDA and some aspects of implementation of the programme.

## MATERIALS AND METHODS

The study was conducted in Burdwan district of West Bengal, India where the MDA has been undertaken since 2005. Burdwan is a district situated in the central part of South Bengal having an area of l7,024 sq. km, with average annual rainfall of 1,460 mm and temperature varying from 5 ^0^C to 36 ^0^C. According to 2001 Census in India, the district had a population of 6,895,514, of whom 48% were female. A cross-sectional descriptive study was conducted in July 2010. As per the guideline suggested by task force of the Government of India for evaluation of filaria elimination programme, four clusters were identified (three from rural and one from urban areas) for the study (7). The purpose of the population-based survey was to provide a coverage estimate that is statistically likely to be representative of the sampled population. The sampling design adopted in the study would provide an estimate of actual coverage to the accuracy of plus or minus 6.5% (8).

The Primary Health Centre (PHC) constituted the sampling frame at the first stage of sampling, and the villages under the PHC constituted the sampling frame at the second stage. As this was a post-MDA sample survey for assessing drug coverage, care was taken to ensure representative response from individuals living in the clusters with variable reported coverage immediately preceding the MDA round. All PHCs in a district were classified into three strata according to reported coverage of MDA activities undertaken during 2010 (PHC area with <50%, between 50% and 80%, and >80% coverage). Total number of PHCs in the district was 106 in 2010. A complete list of the villages was prepared for each of the strata, and one village was selected randomly, using random number table from each stratum. As none of the PHCs reported coverage less than 50% in the previous round, we selected two villages randomly from the stratum with reported 50-80% coverage. For urban area, one of the municipalities was selected randomly from the list of municipalities in the district and, subsequently, one ward was selected randomly from the selected municipality.

For the purpose of the survey, a central point was identified in each of these clusters, and the first house was selected randomly (any number between 1 and 9). The next house was selected having the nearest entrance. Thereafter, a minimum of 30 households were selected consecutively in this manner, covering a minimum of 150 persons from each of the clusters. Finally, 603 individuals from 154 households constituted the study population.

The head of the family or other responsible member present at the time of survey was interviewed with the help of predesigned, pretested semi-structured questionnaire. We collected data on drug distribution, consumption, side-effects following DEC consumption, and awareness of lymphatic filariasis, including MDA programme. Sources of information on MDA programme was assessed among those who knew about MDA programme.

All data were compiled and analyzed applying appropriate statistical method. The assessment was completed within three weeks of completion of the MDA round. Nine drug distributors and other healthcare providers associated with this programme were interviewed to generate data regarding programme implementation.

### Ethical clearance

Ethical clearance was obtained from institutional ethical committee of Burdwan Medical College. The study was carried out on request of the Department of Health and Family Welfare, Government of West Bengal. According to the guideline issued by the Department, informed consent was obtained from the study population. The purpose of the research was communicated to the selected household members, and their oral informed consent was obtained before administering the questionnaire. They were assured of confidentiality and anonymity.

### Analysis

The assessment in this study was made in terms of proportion of people who have actually received DEC tablets (i.e. coverage of drug distribution), those who have ingested the tablets (i.e. consumption of DEC among sampled population), compliance (proportion ingested at sufficient dose of DEC by those who received the tablets) and effective coverage (i.e. proportion ingested at appropriate dose of DEC by eligible individuals) in the selected areas. Coverage and consumption of DEC was further analyzed by age and sex. We entered data on Excel sheet and analyzed with MS Excel software.

## RESULTS

There were 639 individuals in 154 households, of whom only 603 were eligible for administration of MDA at the time of drug distribution. These 603 individuals who were eligible for MDA constituted the study population. A little more than half (52.57%) of the population eligible for MDA was female, and the majority (66.99 %) of population was in the age-group of 15 years and above. Children below two years, pregnant women, and severely-ill persons were excluded. Of 154 families, 134 (87%) received drugs; rest of the families did not receive antifilarial drugs (Table 1).

**Table 1.** Coverage of and compliance with annual single-dose DEC in Burdwan district during 2010

Sex	Eligible population	No. of eligible people given DEC	Drug distribution coverage (%)	No. of people consuming DEC	Compliance (%)*	Effective coverage (%)
Male	286	141	49.30	85	60.28	29.72
Female	317	153	48.26	121	79.08	38.17
Total	603	294	48.76	206	70.07	34.16

χ^2^=0.03 (Drug distribution coverage among males and females), df=1, p=0.86; χ^2^=11.49 (Compliance among males and females), df=1, p=0.00;

*Compliance in percentage=Number of people who had ingested sufficient dose of DEC tablets/Total no. of people who had received the DEC tablets×100; df=Degree of freedom

### Coverage by age

We analyzed the coverage of drug distribution, proportion of non-compliance, and effective coverage among different age-groups (Figure). It was observed that some people received the drugs but did not consume or consumed at suboptimal dose (non-compliance). The proportion of non-compliance varied between 14.59% and 15.69% among different age-groups. The proportion of effective coverage was 41.18% and 20% in 6-14 years and 2-5 years age-group respectively.

### Reasons for non-consumption of drug

Overall, 51.24% of eligible beneficiaries did not receive DEC tablets, and even some of those who received the drugs did not consume DEC in adequate dose (Table 2). No definite reason was stated as a cause of non-consumption in one-quarter of eligible beneficiaries (25.94%). Fear of side-effects (20.15%), lack of awareness of MDA programme (16.88%), absence at the time of drug distribution (14.61%), being not convinced about the benefit of MDA (12.59%) were the common causes of non-compliance. The ‘others’ category includes some trivial reasons, such as ‘forgot to take’, ‘misplaced the drug’, etc.

**Figure.**
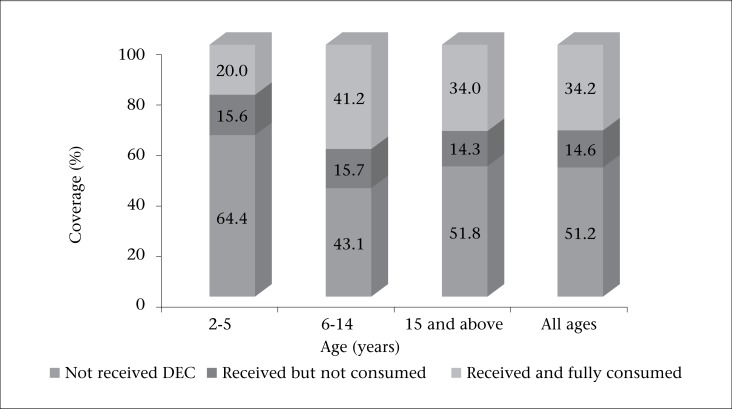
Coverage and consumption of DEC according to age-group

**Table 2.** Major reasons for non-consumption of DEC (n=397)

Reason	Number	Percentage
No definite reasons stated	103	25.94
Fear of side-effects	80	20.15
Do not know MDA	67	16.88
Absent at the time of drug distribution/administration	58	14.61
No faith in MDA	50	12.59
Sickness	14	3.53
Others	25	6.30

### DEC consumption and side-effects

We assessed the side-effects after consumption of DEC among the study population. Of 206 persons who consumed DEC, only 6 (2.91%) reported side-effects. The most common side-effect was dizziness (4), followed by nausea and/or vomiting (3). These appeared within hours of drug intake and disappeared within 2-3 days. In none of the cases, any treatment was taken.

### Awareness of community regarding MDA

Data on awareness could be collected from only 131 families. Only the head or any responsible senior member from each family was interviewed to assess the awareness of community regarding filaria and MDA programme (Table 3).

**Table 3.** Knowledge and perceptions of people about lymphatic filariasis and its elimination programme (n=131)

Knowledge	Number	Percentage
Heard about filariasis	69	52.67
Correct knowledge about at least one sign or symptom of filariasis*	26	19.85
Correct knowledge about transmission of filariasis (mosquito-borne)	20	15.27
Correct knowledge about prevention (Mosquito control, MDA)	23	17.56
Heard of MDA programme	76	58.02

*Periodic fever, swelling of leg (lymphedema, elephantiasis), hydrocele, adenitis

About half of the families (52.67%) were aware of the existence of filaria as a health problem. Less than one-fifth of the study families (19.85 %) could mention correctly at least one presenting sign or symptom of filaria. Most of the families did not know the role of mosquitoes in the transmission of the disease. Few people explained the association between LF and hydrocele or the role of mosquitoes. About 58% of the families were aware of MDA programme in the locality. About 58% respondents knew that drug administration (MDA) was being done in the locality but the majority of them did not know that it was for elimination of LF.

### Sources of knowledge

People were asked about the channel through which they came to know about the MDA programme and which communication method had influenced them. It was observed that majority of respondents (82.89) received information from health personnel through interpersonal channel (Table 4).

**Table 4.** Source of information regarding MDA in Burdwan district (n=76)

Source of information	Number	Percentage
Health personnel	63	82.89
*Panchayat* representatives	12	15.79
Relatives/neighbours	9	11.84
Mass media	21	27.63
Others	30	39.47

Multiple responses accepted

### Some observations on programme implementation

During the coverage survey, we collected additional information on programme implementation in relation to drug distribution component. As per programme guideline, the drug distributors should supervise drug intake to ensure compliance. We conducted in-depth interviews with distributors to collect information on supervised drug intake, timing of drug distribution and the information provided to recipients at the time of drug distribution. Altogether, nine drug distributors worked in the study area; they visited each family only once during daytime. They distributed drugs to individuals present during the visit and handed over the DEC to relatives for those who were absent. Seven out of nine drug distributors advised the beneficiaries to take the drugs after meal. The drug distributors said they could not cover all beneficiaries because of lack of interest among the people and absence at the time of drug distribution.

## DISCUSSION

Although the coverage of drug distribution was 48.76%, the effective coverage was 34.16% only. Effective coverage rate is the product of coverage by the health system and compliance in the community. A sustainable high coverage of 85% or more is required for stopping transmission and elimination of disease from the community (4). Other researchers in West Bengal reported more or less similar findings on coverage (9). However, coverage rate reported by other researchers was comparatively higher than the coverage reported in the present study (10,11). The current approach of drug delivery has been found to achieve an effective coverage of 34.16% only. There is an urgent need for more effective drug-delivery strategies that are adapted to local need.

The proportion of non-compliance varied between 14.25% and 15.69% among different age-groups. In the present study, 14.59% of population failed to consume the drug even after receiving it, which was more or less similar (around 11%) to that reported in another study (10).

It was stated by drug distributors that they have visited each family only once to distribute the DEC. Repeated contacts may be required to increase the coverage. To cover the absentees and poorly-covered areas, there is provision of ‘mop-up’ rounds but this activity has not been carried out to maximize the drug consumption. This indicates lack of supervisory activity for the programme. In majority of cases, the DEC intake was not supervised. Similar findings were reported by P. Ray Karmakar *et al*. (9) and Mahalakshmy T *et al*. (11).

In the present study, the main reasons for non-coverage were inability of workers to cover entire population, not administering drug to unwilling persons or due to misclassification of persons rendering them not eligible. The important causes of non-compliance were non-supervised drug administration and fear of side-effects. The tablets were distributed during the daytime when most people go out for work, leading to the insufficient coverage. The time for the tablet distribution should be the evening to make it convenient for the community. Seven out of nine drug distributors said they thought it was preferable to consume the drugs after meal, and they advised so. This indicates there is need for training before launching the drug distribution programme. A significant proportion of people did not state any definite reason for non-consumption, indicating lack of motivation.

### Side-effects

Drugs were well-tolerated, and side-effects were negligible. Side-effects reported by other researchers were more or less similar as reported in the present study (9). Although the side-effects were insignificant, fear of side-effects was a major cause of non-compliance. Therefore, it is essential to deliver appropriate health information to address people's concerns and fears about the intervention and make arrangement for the management of cases.

### Awareness

The knowledge gap with regard to the disease and prevailing attitudes and perceptions toward the programme may be a major factor for lower compliance. The awareness about MDA activity was limited among 58% of families, and among them, the major source of information was health staff. Similar findings have been reported from other studies in India (9,12).

Effective community mobilization activities are essential to strengthen the people's knowledge and to change their perceptions regarding LF. The investigators probed for the participation of the community people in the programme. It was observed that neither the local authorities sought active help or cooperation of the community members nor they had idea about how to involve them in the programme. Lahariya and Mishra also noted similar findings with regard to community participation in their study (13). Involvement of and coordination with other sectors, involvement of NGOs, local leaders, and self-help groups need to be emphasized. An effective health education campaign to make the community aware about LF and increase their participation in the programme is essential to achieve desired success. The focus of the health education should be on locally-appropriate media and announcements by loudspeakers.

It was observed from the study that drug distributors hardly insisted on supervised ‘on-the-spot’ administration of drugs. Therefore, supervised drug intake was nil or poor in the area. Most respondents who consumed the drug took it after meal; this resulted in poor compliance with drug intake.

The implementation of the programme can be improved by making efficient microplans, ensuring improved supervision, emphasizing the proper training of workers and supervised ‘on-the-spot’ DEC consumption.

### Limitations

Successful implementation of MDA depends on various preparatory activities, such as selection and availability of health staff and/or volunteers, orientation and training of personnel, mobilization of resources, political commitment, advocacy, and social mobilization. However, in the present study, these components could not be assessed due to resource and time constraints. As several rounds of MDA have already been implemented for the last five years, it is now the time to evaluate the impact of the programme. The prevalence and density of microfilariae, together with drug coverage, are currently the best indicators for measuring the impact of MDA. Research on these issues is needed to explore the current status of the problem and whether or not to continue MDA.

### Conclusions

This evaluation study noted that MDA is restricted to tablet distribution only. The major issues of implementation in compliance, health education, fear of side-effects, motivation/promotion measures, and community participation were not being given due attention. The implementation activities should be strengthened immediately in the MDA programme in India to achieve the goal of LF elimination by 2015.
